# Food Biotechnology Potential of Grape-Derived *Aureobasidium pullulans*: Characterization and Screening for Enzyme Production Capacity

**DOI:** 10.3390/foods15091573

**Published:** 2026-05-03

**Authors:** Vesna Milanović, Ana Boban, Federica Cardinali, Andrea Osimani, Lucia Aquilanti, Cristiana Garofalo, Giorgia Rampanti, Irena Budić-Leto

**Affiliations:** 1 Dipartimento di Scienze Agrarie, Alimentari ed Ambientali, Università Politecnica delle Marche, via Brecce Bianche, 60131 Ancona, Italy; v.milanovic@staff.univpm.it (V.M.); a.osimani@staff.univpm.it (A.O.); l.aquilanti@staff.univpm.it (L.A.); c.garofalo@staff.univpm.it (C.G.); g.rampanti@staff.univpm.it (G.R.); 2 Institute for Adriatic Crops and Karst Reclamation, Put Duilova 11, 21000 Split, Croatia; ana.boban@krs.hr (A.B.); irena.budic-leto@krs.hr (I.B.-L.)

**Keywords:** yeast-like fungus, phenotypic screening, hydrolytic enzymes, biogenic amines, stress tolerance, phosphate solubilisation, food bioprocessing

## Abstract

*Aureobasidium pullulans* is a polyextremotolerant yeast-like fungus increasingly recognized for its role in food ecosystems and its emerging potential in flavour development and nutrient modulation. However, systematic evaluations of autochthonous grape-associated populations integrating technological performance and safety-related traits remain limited. This study provides a broad phenotypic screening of 70 isolates from Maraština grapes (Dalmatia, Croatia), applying an integrated functional screening approach to link enzymatic potential, environmental resilience, and food safety. Most isolates displayed multiple hydrolytic enzymes, with widespread cellulase, pectinase, xylanase, esterase, and protease activities. Several isolates showed very high enzymatic indices, supporting their potential for plant-derived substrate transformation, aroma release, and food processing applications. β-glucosidase and urease activities were common, while amylase was limited. Ecological screening confirmed robust adaptability to salinity, osmotic stress, and wide pH ranges. Notably, 31% of isolates demonstrated phosphate solubilization capacity, indicating a possible contribution to mineral bioavailability and nutritional enhancement. Safety screening revealed decarboxylation of selected amino acids, while two isolates lacked detectable activity, highlighting them as candidates for further safety evaluation. Overall, this work establishes a framework for selecting *A. pullulans* isolates for next-generation, flavour-oriented and nutritionally enhanced food applications, supporting sustainable bioprocessing and future industrial validation.

## 1. Introduction

Microorganisms represent a rich reservoir of industrially relevant enzymes and bioactive compounds. Among these, *Aureobasidium pullulans* is a ubiquitous, polymorphic fungus that thrives in diverse environments, including extreme habitats [1,2,3,4]. Its ability to transition between yeast-like, hyphal, and chlamydospore forms facilitates both environmental adaptability and the production of varied secondary metabolites [3,5]. Although traditionally studied for its high-yield production of the polysaccharide pullulan [4,6,7], recent research emphasizes its broader biotechnological utility in food and fermentation processes [1,2,3,4,5,6]. *A. pullulans* can synthesize a diverse array of enzymes, including amylases, cellulases, xylanases, proteases, β-glucosidases, and ureases, which are relevant for sustainable and cost-effective bioprocessing [3,5,8]. For instance, proteases are used in protein modification and cheese production [9], esterases contribute to flavor development [10], and β-glucosidases are involved in aroma release and biomass conversion [11,12]. Pectinases, cellulases, and xylanases are applied in juice processing and plant material transformation [13,14,15], while ureases influence nitrogen metabolism and may contribute to food safety in fermented products [16].

The ability of *A. pullulans* to produce enzymes active across a broad range of pH and temperature conditions highlights its potential for food processing applications, particularly as a sustainable alternative to conventional chemical treatments [5]. Despite the recognized functional diversity of *A. pullulans*, most studies have focused on individual strains or specific applications [17,18]. Although its enzymatic capabilities are well documented [5,8], integrated data linking enzymatic performance, environmental resilience, and safety traits within the same strain collection remain limited, particularly for autochthonous populations from food-related environments. This lack of systematic, strain-level evaluation hinders the identification of robust candidates combining desirable technological properties with adequate safety profiles. Furthermore, the high variability within *A. pullulans* populations highlights the need for integrated approaches that simultaneously assess multiple functional and safety-related characteristics.

Accordingly, this study investigates 70 *A. pullulans* isolates from Maraština grapes collected across 11 vineyards in Dalmatia, Croatia, with the objective of performing a broad phenotypic screening to explore their functional diversity and identify strains with promising technological potential while considering preliminary safety-related traits. Each isolate was evaluated for its ability to produce key hydrolytic enzymes relevant to food processing, including cellulase, xylanase, amylase, pectinase, β-glucosidase, protease, urease, and esterase, as well as for phosphate-solubilizing capacity and biogenic amine production, an important safety consideration in fermented foods and beverages. Their tolerance to high salinity, elevated temperatures, osmotic stress, and a wide pH range was also assessed to determine their robustness under processing conditions.

By integrating these traits within a single collection of autochthonous isolates, this work provides a structured phenotypic dataset that contributes to addressing the current lack of comparative, strain-level studies and supports the identification of candidate strains for subsequent in-depth characterization and validation in food systems.

## 2. Materials and Methods

### 2.1. Aureobasidium pullulans Isolates

The 70 *A. pullulans* isolates used in this study were previously obtained from Maraština grapes collected in 11 vineyards across the winegrowing region of Dalmatia (Croatia), covering the subregions of Northern Dalmatia (ND), Dalmatian Hinterland (DH), and Central and Southern Dalmatia (CSD). The vineyards DR (Dračevica), K (Kruševo), P (Prapatna 1), and B (Prapatna 2) were located on the island of Korčula. These, along with VP (Kaštela) and I (Institute for Adriatic Crops and Karst Reclamation, Split), are part of the CSD subregion. The O (Oklaj) vineyard belonged to the DH subregion, while the remaining vineyards, including S (Smilčić), N (Polača), Z (Stankovci), and V (Vukšić), were situated in the ND subregion. The overall sampling area spanned approximately 177 km between the northmost (vineyard S) and southmost sites (island of Korčula), reflecting a broad geographical gradient [19,20].

The collected grape berry samples were homogenized, serially diluted in sterile physiological saline solution (0.85% *w*/*v* NaCl), and plated (0.1 mL) on Wallerstein Laboratory (WL) differential agar. After incubation at 25 °C for 5 days, colonies were selected based on their morphology and purified by subculturing [20]. Molecular identification of the isolates was carried out by sequencing the ITS1–5.8S–ITS2 rDNA region, followed by Basic Local Alignment Search Tool (BLAST+ 2.17.0) analysis [21], as described by Milanović et al. [20]. The isolates were stored at −80 °C in a 1:1 mixture of Yeast extract–Peptone–Dextrose (YPD) broth (10 g/L yeast extract, 20 g/L peptone, 20 g/L dextrose) and glycerol, as part of the yeast collection of the Institute for Adriatic Crops (I, Split, Croatia). Unless otherwise specified, all chemicals and reagents used in this study were of analytical grade and purchased from Thermo Fisher Scientific (Basingstoke, UK).

### 2.2. Characterisation of Aureobasidium pullulans Isolates

Prior to analysis, the isolates were initially cultured in Malt Extract Broth (MEB) and incubated at 25 °C for 72 h. The cells were harvested by centrifugation (Rotofix 32 A, Hettich, Tuttlingen, Germany) at room temperature for 5 min at 4000 rpm (2683 × *g*). After discarding the supernatant, the cell pellets were washed twice with sterile physiological saline solution and finally resuspended in 10 mL of the same solution. Cell concentration was determined by measuring absorbance at 600 nm using a UV–Vis spectrophotometer (UV-1800, Shimadzu Corporation, Kyoto, Japan). Samples were subsequently diluted in the same solution to a final concentration of 6 Log CFU/mL, determined spectrophotometrically using a previously established calibration curve correlating OD_600_ values with viable cell counts, and verified by plate counting on Malt Extract Agar (MEA). The prepared suspensions were spot-inoculated (5 µL) onto specific agar media for each test. All tests were conducted in triplicate.

#### 2.2.1. Halotolerance

To evaluate the NaCl tolerance of the isolates, 5 µL aliquots of cell suspensions were applied onto YPD agar plates containing increasing concentrations of NaCl (0, 2, 4, 6, 8, 10, 12, 14, 16, and 18% *w*/*v*). The plates were incubated at 25 °C for 72 h. Salt tolerance was determined as the highest NaCl concentration that supported visible growth of each isolate, following the approach described by Pinto et al. [22].

#### 2.2.2. Osmotic Tolerance

Osmotic tolerance of *A. pullulans* isolates was assessed by spotting 5 µL of cell suspensions onto YPD agar plates supplemented with 30% and 50% glucose. Following incubation at 25 °C for 48 h [23], growth was evaluated and recorded as either positive (+) or negative (–) for each glucose concentration.

#### 2.2.3. pH Tolerance

To assess pH tolerance, 5 µL of cell suspensions were spotted onto YPD agar plates adjusted to pH 4, 5, 6, 7, 9, and 11, and incubated at 25 °C for 48 h [22]. Tolerance was recorded as positive (+) or negative (–) for each tested pH value.

#### 2.2.4. High Temperature Tolerance

Tolerance of the isolates to high temperatures was evaluated by monitoring their survival after exposure to 50 °C for various time intervals, as previously described by Zajc et al. [24] with slight modifications. Briefly, cell suspensions from actively growing cultures in MEB were adjusted to approximately 6 Log cells/mL. Aliquots (1 mL) of each culture were incubated at 50 °C for 2, 4, 6, and 24 h. At each time point, 5 μL of the heat-exposed suspension was spot-inoculated onto MEA plates, which were subsequently incubated at 25 °C for 72 h. Thermal tolerance was defined as the longest exposure time (in hours) at which visible growth was observed post-incubation.

#### 2.2.5. Phosphate Solubilization

The ability of the isolates to solubilize phosphate was assessed using Pikovskaya medium, composed of glucose (10 g/L), NaCl (0.2 g/L), (NH_4_)_2_SO_4_ (0.5 g/L), Yeast extract (0.5 g/L), MnSO_4_ (0.1 g/L), MgSO_4_ (0.1 g/L), Agar (20 g/L), and Ca_3_(PO_4_)_2_ (5 g/L), which was sterilized separately. The plates were incubated at 25 °C for 10 days [22]. Phosphate solubilization was determined based on the presence (+) or absence (−) of a clear halo surrounding the colony.

#### 2.2.6. Production of Biogenic Amines

To evaluate the capacity of the isolates to produce biogenic amines, selective media specific for each of the tested amino acids (histidine, phenylalanine, ornithine, and lysine; Sigma-Aldrich, St. Louis, MO, USA) were prepared. Each medium contained glucose (0.1 g/L), bromocresol purple (0.06 g/L), agar (1.5% *w*/*v*), and 10 g/L of the respective amino acid. Following autoclaving, a filter-sterilized yeast nitrogen base solution (6.7% *w*/*v*) was added aseptically at a ratio of 100 mL/L. The final pH of the medium was adjusted to 5.3 ± 0.02 using HCl. Isolates (5 μL) were spot inoculated onto the surface of the agar plates and incubated at 25 °C for 4 days. A positive result, indicative of amino acid decarboxylation and subsequent biogenic amine production, was identified by the formation of a violet halo around the colonies on the yellow medium, as described by Bavaro et al. [25].

#### 2.2.7. Enzymatic Activities

Cellulolytic and xylanolytic activities were evaluated using media described by Zajc et al. [2] with slight modifications. The cellulase medium (pH 5.2) contained 1.0 g/L NH_4_H_2_PO_4_, 0.2 g/L KCl, 0.2 g/L MgSO_4_·7H_2_O, 0.2 g/L CaCl_2_, 40 g/L carboxymethyl cellulose (CMC), and 15 g/L agar dissolved in deionized water. For the xylanase assay, the same basal medium composition was used, except that 10 g/L xylan replaced the CMC and the pH was adjusted to 4.2. Following inoculation, plates were incubated at 25 °C for 8 days. Enzymatic activity was detected by flooding each plate with 5 mL of Lugol’s iodine solution (Sigma-Aldrich) diluted 1:1 with distilled water, allowing it to stand for two minutes, then rinsing with deionized water. The formation of a clear halo surrounding the colonies against the dark background was interpreted as evidence of enzymatic degradation of the respective substrate.

To evaluate amylolytic activity, the strains were inoculated onto potato dextrose agar (PDA) supplemented with 10 g/L soluble starch. Plates were incubated at 24 °C for 8 days. After incubation, each plate was flooded with iodine solution and allowed to react for 2 min to visualize starch degradation. The presence of clear halos surrounding the colonies against the dark background of the stained medium was considered indicative of amylolytic activity.

Pectinolytic activity was evaluated by inoculating the strains onto nutrient agar (NA) composed of 5 g/L peptone, 3 g/L beef extract, 5 g/L NaCl, and 18 g/L agar, adjusted to pH 6.8, and supplemented with 5 g/L pectin [22]. Plates were incubated at 28 °C for 48 h, after which they were flooded with iodine solution and left to react for 2 min. The presence of clear halos surrounding the colonies against the dark background of the stained medium was considered indicative of pectinase activity.

Screening for *β*-glucosidase activity was performed on Esculin agar following the protocol by Mateo [26] with minor modifications. The medium consisted of 2 g/L glucose, 1 g/L peptone, 1 g/L yeast extract, 0.3 g/L esculin, 1 g/L C_6_H_11_FeNO_7_, and 18 g/L agar. Plates were incubated at 30 °C for 3 days. Enzymatic activity was indicated by the formation of a dark halo surrounding the *A. pullulans* colonies.

Urease activity was assessed using a urea-containing medium [2,24] prepared as follows: the basal medium consisted of 1.0 g/L NH_4_H_2_PO_4_, 0.2 g/L KCl, 0.2 g/L MgSO_4_·7H_2_O, 0.05 g/L bromcresol purple, and 10 g/L glucose, solidified with 18 g/L agar in deionized water. A sterile aqueous urea solution (500 g/L) was filter-sterilized and added aseptically to the autoclaved basal medium to achieve a final urea concentration of 20 g/L. Both control plates (without urea) and test plates (with urea) were inoculated. Strains were considered positive for urease activity if control plates remained yellow, indicating no pH change, while test plates showed a color shift from red to purple, reflecting an increase in pH due to urea hydrolysis.

Protease activity was assessed as described by Boban et al. [27] by spotting *A. pullulans* cell suspensions onto Petri dishes containing a medium prepared by mixing two separately formulated components: (i) a solution of yeast extract (3 g/L), malt extract (3 g/L), glucose (10 g/L), NaCl (5 g/L), peptone (5 g/L), and agar (20 g/L), with the pH adjusted to 3.5 using 0.1 M HCl; and (ii) a 10% (*w*/*v*) skim milk solution preheated at 100 °C for 10 min. The plates were incubated at 25 °C for three days. Protease activity was scored as positive when a clear halo was visible around the isolate growth.

For esterase activity testing, the isolate suspensions were applied onto Petri dishes containing a medium consisting of peptone (10 g/L), NaCl (5 g/L), Tween 80 (10 g/L), CaCl_2_·2H_2_O (0.1 g/L), and agar (20 g/L), with the pH adjusted to 6.8 as described by Boban et al. [27]. The plates were incubated at 25 °C for six days. Esterase production was evaluated by observing the formation of an opaque precipitate surrounding the colony.

The activities of amylase, cellulase, pectinase, and xylanase were assessed by calculating the enzymatic index (EI), defined as the ratio between the diameter of the clearing zone and the diameter of the colony, both measured in millimeters using a millimeter-scale ruler. The activities of *β*-glucosidase, urease, protease, and esterase were evaluated qualitatively and recorded as either positive (+) or negative (−) reactions.

### 2.3. Statistical Analysis

For the analysis of the EIs (Table 1) and halotolerance (Table 2) of *A. pullulans* isolates across vineyards, one-way analysis of variance (ANOVA) was performed, followed by post hoc comparisons using the Tukey–Kramer HSD test (*p* < 0.05) to estimate significant differences. Statistical analyses were conducted using IBM^®^ SPSS^®^ Statistics for Windows, version 23.0 (SPSS Inc., Chicago, IL, USA). In addition, qualitative traits (recorded as positive/negative) were summarized descriptively as frequencies and percentages and were not subjected to inferential statistical analysis.

## 3. Results and Discussion

**Figure 1:**
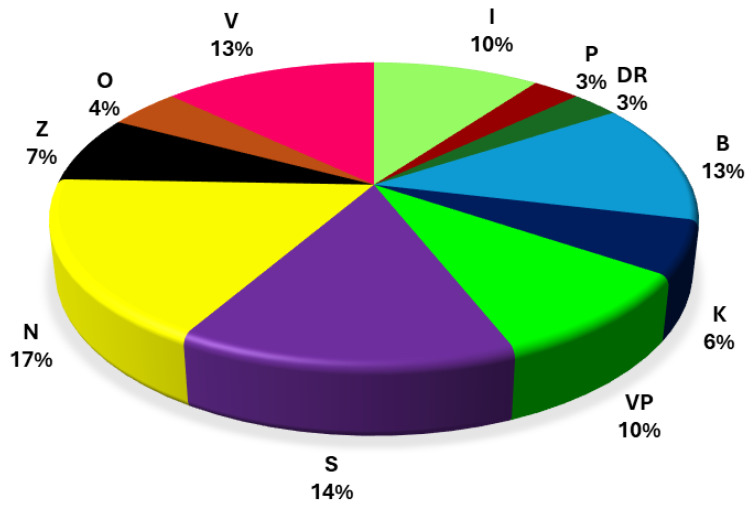
Relative distribution of *Aureobasidium pullulans* isolates among vineyards.

The relative distribution of isolates by vineyard is shown in Figure 1. The lowest portion (3%) of the isolates was recovered from the vineyards P and DR, both from the CSD subregion, particularly from the Korčula island, whereas the highest proportion of the isolates was recovered from the N vineyard (ND subregion), 17%. The relatively even presence of *A. pullulans* across geographically distant locations confirms its ubiquity in vineyard ecosystems, consistent with previous work describing this species as a dominant and persistent colonizer of grape surfaces and other plant hosts in diverse climates [8,28]. Such persistence is attributed to its polyextremotolerant nature, versatile metabolism, and ability to form protective melanized cell walls, which enhance survival under fluctuating vineyard conditions [3].

The enzymatic screening revealed that *A. pullulans* isolates from all vineyards exhibited multiple hydrolytic activities, although the intensity and prevalence varied geographically, suggesting differential adaptation of isolates to local environmental conditions and substrate availability. The Dalmatian subregions are characterized by a Mediterranean climate with local variability in temperature and precipitation. The CSD region exhibits higher average temperatures (18.0 °C) and slightly greater daily precipitation (6.4 mm), whereas ND (16.8 °C) and DH (15.4 °C) are comparatively cooler and drier (6.0 and 5.8 mm, respectively). In addition, vineyards differ in soil composition (e.g., red soils, loam, sand, and karst-derived substrates), altitude, and proximity to the sea, which may influence moisture availability and nutrient dynamics [19]. These environmental factors are known to shape vineyard-associated microbial communities and their functional traits, potentially contributing to the variability observed in enzymatic profiles among isolates [19,20]. Supplementary Table S1 shows the results of the screening for enzyme activities, expressed as EI for amylase, cellulase, pectinase, and xylanase, or as positive/negative for the other enzymes. Table 1 presents the mean EI values for isolates from each vineyard, together with the number of positive isolates out of the total isolates for enzymes recorded as either positive or negative.

Amylases catalyse the hydrolysis of α-1,4-glycosidic bonds in starch, yielding maltose, glucose, and dextrins. They are among the most widely applied enzymes in food processing, particularly in brewing, baking, and syrup manufacture, where they facilitate starch conversion into fermentable and functional sugars [29]. In addition to their role in the food industry, amylases also contribute to non-food applications, including in the textile, detergent, pharmaceutical, and biofuel industries [30]. In *A. pullulans* isolates from Maraština grapes, amylase activity was not uniformly present, with 27 isolates (39%) showing no detectable activity (EI = 1.00). No significant differences in EI were observed among vineyards, except for vineyard N (ND subregion), which displayed the highest average EI (1.26). In contrast, isolates from vineyard P exhibited no detectable amylase activity. Notably, isolate N-1 demonstrated the maximum EI value (2.00), comparable to or exceeding values reported for some of the most active strains described previously [22,24]. However, it is important to note that the EI is a semi-quantitative measurement influenced by colony growth rates and substrate diffusion in agar. Thus, while N-1 is a promising candidate, its industrial efficiency must be confirmed through quantitative activity assays. The generally low amylase activity observed in the present study is consistent with reports that *A. pullulans* typically shows strong pectinolytic and cellulolytic activity but low or undetectable α-amylase activity [31].

Cellulases hydrolyze *β*-1,4-glycosidic bonds in cellulose, producing glucose and oligosaccharides, and are important industrial enzymes for biomass conversion, textiles, paper, and food sectors [32]. In our *A. pullulans* isolates, cellulase activity was detected in all samples but varied significantly across vineyards. The isolates from vineyard Z exhibited the lowest average EI value (2.22), whereas those from vineyard DR showed the highest mean EI (3.92). Among individual isolates, S-3 stood out with the highest EI of 5.33. Overall, the cellulase EI values recorded in the present study are higher than those reported previously for *A. pullulans* isolates [2,24], except for strain Fito_F278, which displayed an exceptionally high EI of 10.50 [22].

Pectinases catalyse the hydrolysis of pectin, a complex polysaccharide of plant cell walls, into simpler molecules such as galacturonic acid. These enzymes play a key role in food processing, particularly in fruit juice and wine clarification, where they enhance juice yield, reduce viscosity, and facilitate filtration by promoting cell wall disassembly [33]. Unlike amylases and cellulases, which act on starch and cellulose, respectively, pectinases specifically target pectic substances, enabling tissue softening and the release of intracellular components, a property that is essential for the efficient processing of fruit-based foods [34]. In our *A. pullulans* isolates, pectinase EI values were generally homogeneous across vineyards, ranging from a mean of 2.63 for isolates from vineyard DR to 3.78 for isolates from vineyard VP. Notably, isolate S-2 exhibited the highest EI of 5.38. These results indicate that pectinase production in *A. pullulans* is relatively stable across geographic locations, although certain strains can produce exceptionally high levels. The pectinase EI values observed in the present study are more than twice as high as those reported in previous studies for *A. pullulans* isolates [2,24], though they are considerably lower than the exceptionally high EI of 10.00 reported for strain Fito_F278 [22]. While these plate-based results identify interesting candidates for industrial processes requiring pectin degradation, the actual performance of these enzymes in complex food environments, where pH and temperature fluctuations occur, remains to be validated.

Xylanases catalyse the hydrolysis of *β*-1,4-xylosidic linkages in xylan, the principal hemicellulosic component of plant cell walls, releasing xylo-oligosaccharides and xylose. They are used in pulp bleaching to improve dough handling and bread quality in baking through the modification of cereal fiber components and in animal feed to enhance nutrient accessibility and digestibility [14,35]. Among the vineyards, the average xylanase EI values ranged from 1.34 in vineyard I (CSD subregion) to 3.39 in vineyard V (ND subregion). At the single isolate level, only two isolates lacked detectable xylanase activity, whereas the highest EI values were recorded for isolates V-12 (6.00), S-4 (4.80), and V-8 (4.40), all originating from ND vineyards. These EI values are markedly higher than those previously reported for *A. pullulans* [2,24], suggesting that these grape-associated isolates may possess specialized hemicellulolytic systems. Nevertheless, their practical utility in baking or byproduct processing warrants further investigation into their substrate specificity and thermal stability.

*β*-glucosidases hydrolyse terminal non-reducing *β*-D-glucosidic linkages, releasing glucose from cellobiose and other *β*-linked oligosaccharides. They play critical roles in cellulose degradation, aroma liberation in wines, and bioconversion of glycosides [36]. Most isolates in this study (58 out of 70) exhibited *β*-glucosidase activity. All isolates from vineyards I, P, DR, K, Z, and O were *β*-glucosidase positive, whereas the lowest proportion of active isolates was observed in vineyard S, with only 3 out of 10 showing detectable activity. *β*-glucosidase activity is a common feature of *A. pullulans*, which is known to produce diverse glycosidases involved in the hydrolysis of plant-derived glucosides and aroma precursors [8,12]. *A. pullulans* β-glucosidase has been demonstrated to substantially increase monoterpene concentrations (e.g., linalool, geraniol) in treated wines [37]. The widespread presence of this activity in our isolates supports their ecological role in degrading plant glycosides, but their specific impact on aroma development is highly dependent on the enzyme’s affinity for specific precursors and its activity under low pH conditions typical of many fruit juices [38].

Esterases catalyse the hydrolysis of ester bonds, releasing alcohols and acids from a wide range of natural and synthetic substrates. These enzymes are essential for lipid turnover and detoxification in microorganisms and have diverse industrial applications in flavor development, pharmaceuticals, detergents, and biodiesel synthesis [39]. In the food and beverage sector, these enzymes influence flavour by generating ester compounds responsible for fruity and floral aromas. Esterases have been utilized to tailor flavour profiles in fruit juices and fermented beverages by converting aroma precursors, such as grape-derived fatty acids, into volatile esters [40]. All *A. pullulans* isolates analysed in this study exhibited esterase activity. Such ubiquity aligns with previous reports demonstrating that esterase activity is among the most stable enzymatic features of the species, regardless of environmental origin or substrate availability [2,5,8,24].

Proteases catalyze the hydrolysis of peptide bonds and are widely applied in food processing, including cheese and yogurt production, meat tenderization, and dough conditioning, as well as in non-food industries such as detergents and leather treatment [41]. In the present study, protease activity was detected in the majority of *A. pullulans* strains, except for four isolates originating from vineyards K and S. The widespread occurrence of proteolytic activity among *A. pullulans* strains has been consistently documented in previous studies [2,5,8,24,41], further supporting the biotechnological relevance of this species.

Ureases catalyse the hydrolysis of urea into ammonia and carbon dioxide, contributing to nitrogen metabolism and offering applications in agriculture, diagnostics, and bioremediation [42]. In winemaking, urease activity is particularly relevant because urea can serve as a precursor for ethyl carbamate formation, a potentially harmful compound in fermented beverages [43]. In our *A. pullulans* isolates, urease activity was detected in 33 of 70 strains, with the highest prevalence in vineyard N (67% of isolates positive), while no activity was observed in isolates from vineyards P and Z. These findings are consistent with previous reports, which showed generally moderate urease activity in some *A. pullulans* strains [2,24] or no detectable activity in others [8,22].

Environmental stress factors such as salinity, osmotic pressure, temperature, and pH play a decisive role in shaping the growth and functional traits of *A. pullulans*. The results of the screening conducted in this study are summarized in Table 2, with detailed data provided in Supplementary Table S2. While these laboratory stress-response profiles provide a baseline for selection, the transition from plate-based tolerance to effective performance in real application systems involves complex biotic and environmental interactions that are not captured in this screening.

Among these traits, halotolerance is a key adaptive feature that enables survival under diverse environmental conditions and supports applications in food biotechnology, including fermentation and preservation processes. Furthermore, halotolerance is associated with cross-protection against oxidative, heavy metal, and thermal stresses, potentially enhancing the effectiveness of *A. pullulans* as a biocontrol agent in food-related and agricultural systems [24,44]. In our study, most isolates from the CSD vineyards grew well in the presence of 14% NaCl, with the sole isolate I-19 able to grow even at 16%. In contrast, isolates from the ND and DH vineyards were more susceptible to salt stress, generally tolerating concentrations between 10–12%, with a few strains inhibited already at 6% NaCl. These findings confirm the broad halotolerance previously reported for *A. pullulans* [24], although the maximum tolerance observed in our collection was slightly lower than the 18% NaCl reported in that study, which remains the highest concentration so far documented for this species. The variability among subregions suggests that local vineyard conditions may shape strain-specific tolerance levels. The higher halotolerance observed in CSD isolates could reflect adaptation to coastal environments where salinity and osmotic fluctuations are more pronounced, while the reduced tolerance in ND and DH isolates may be linked to inland microclimates with lower salt exposure. Such ecological differentiation highlights the plasticity of *A. pullulans* and its ability to adjust to local stress regimes.

Furthermore, our *A. pullulans* isolates showed strong resistance to osmotic stress; all strains grew at 30% glucose, while most tolerated 50%, with only a few from vineyards originating from ND failing under this condition This high-level tolerance, largely conserved across vineyards, reflects efficient osmotic regulation mechanisms, including compatible solute accumulation and melanized cell walls, which support survival on grape surfaces and enhance their biotechnological potential in high-sugar food processes [1,3,8]. Notably, most isolates unable to grow at 50% glucose were also characterized by lower halotolerance (≤10% NaCl), suggesting a partial correlation between osmotic and salt tolerance under low water activity conditions.

As regards high-temperature tolerance, the isolates exhibited limited resistance. Survival at 50 °C was detected only after short exposure (2 h) and only in a few strains, primarily those originating from the ND and DH vineyards. No isolate survived 4 h at 50 °C, confirming that thermal tolerance is not a dominant trait in this population. These findings partially align with Zajc et al. [24], who observed that most *A. pullulans* strains survived 2 h at 50 °C, whereas only a minority tolerated 4–6 h and just two strains recovered after 24 h. Such limited thermotolerance restricts their suitability for high-temperature industrial processes, although it remains compatible with applications conducted under moderate thermal conditions, including winemaking and postharvest biocontrol.

Regarding the resistance of the isolates to pH variations, our results show that most isolates were able to grow across a wide pH range, from 4 to 11, with only two isolates exhibiting restricted growth. Similar trends have been reported in the literature, describing *A. pullulans* as polyextremotolerant and capable of maintaining cellular homeostasis under pH stress through metabolic adjustments involving amino acid and carbohydrate pathways [45]. This physiological flexibility not only supports its ecological success but also underpins its potential for diverse biotechnological applications, where tolerance to pH fluctuations can be advantageous for enzyme production, polysaccharide synthesis, and other industrial processes.

Phosphorus is an essential macronutrient for plants, participating in numerous metabolic processes, and its deficiency can cause severe physiological disorders. In soils, however, phosphorus is often present in insoluble forms, limiting its bioavailability. Microorganisms capable of solubilizing phosphate play a vital role in nutrient cycling and plant growth promotion, offering a sustainable alternative to chemical fertilizers as biofertilizers [46]. In our study, phosphate solubilization was observed in 31% of *A. pullulans* isolates (Table 2; Supplementary Table S2), in contrast to previous reports where nearly all tested strains exhibited this trait [47]. Clear differences were observed among vineyards; the highest frequencies of positive strains occurred in vineyard V (89%), followed by Z (60%) and K (50%), whereas no activity was detected in isolates from vineyards P, DR, and VP, all within the CSD subregion. Overall, isolates from the ND subregion showed greater phosphate-solubilizing capacity compared with other subregions. This variability suggests that phosphate solubilization is a strain-specific trait influenced by local vineyard conditions and selective pressures. Beyond agricultural benefits, these traits have implications for the food industry. *A. pullulans* strains capable of mobilizing nutrients can enhance the quality and yield of raw plant materials used in food processing and fermentation, supporting both sustainable production and industrial applications as a source of enzymes and bioactive compounds.

Biogenic amines (BAs) such as histamine, tyramine, putrescine, and cadaverine are important for wine safety because they can cause adverse effects in consumers, particularly when combined with ethanol [48,49]. Although lactic acid bacteria are the main producers, several studies have shown that *Saccharomyces cerevisiae* and various non-*Saccharomyces* yeasts can also synthesize BAs during fermentation [50,51,52]. To the best of our knowledge, *A. pullulans* has often been regarded as safe, with no evidence of BA production reported. However, safety should be evaluated at the strain level, as variability in traits such as biogenic amine production may occur. In contrast, screening of 70 *A. pullulans* isolates from Maraština grapes revealed a widespread capacity to decarboxylate amino acids (Table 2; Supplementary Table S2). Histidine, phenylalanine, and ornithine were frequently decarboxylated, whereas lysine was never decarboxylated, indicating the absence of cadaverine synthesis. In CSD vineyards such as I, P, DR, and K, 100% of isolates tested positive for histidine decarboxylation, whereas ND and DH sites were more variable. Notably, all isolates from vineyard I were able to decarboxylate histidine, phenylalanine, and ornithine. Conversely, two isolates from vineyard B (B-21 and B-24) did not decarboxylate any tested amino acids, which could indicate their potential as low-risk strains for biotechnological applications.

## 4. Conclusions

This study provides a systematic phenotypic assessment of *A. pullulans* populations associated with Maraština grapes, revealing a previously underexplored reservoir of functional diversity within a single viticultural ecosystem. By integrating enzymatic profiling, stress tolerance, phosphate-solubilizing capacity, and biogenic amine screening across 70 isolates, this work offers a comprehensive strain-level evaluation of grape-derived *A. pullulans* relevant to food microbiology. The widespread occurrence of cellulase, pectinase, xylanase, esterase, protease, and β-glucosidase activities, together with enzymatic indices exceeding values commonly reported for this species, suggests a notable lignocellulolytic potential of vineyard isolates and indicates their possible applicability in juice processing, maceration, flavor enhancement and aroma release, as well as agro-industrial by-product valorisation. Several isolates emerged as particularly promising candidates for targeted applications; isolate N-1 exhibited the highest amylase activity, while S-3 and S-2 showed the highest cellulase and pectinase activities, respectively. The xylanolytic isolate V-12 demonstrated potential for lignocellulosic biomass processing. However, these findings are based on plate-based and semi-quantitative assays, and further validation under process-relevant conditions is required. The pronounced tolerance to osmotic stress, salinity, and variable pH further confirms the ecological plasticity of these strains and suggests potential robustness under technologically relevant conditions. In particular, isolate I-19, which tolerated up to 16% NaCl, represents a notable example of high salinity resistance. Additionally, the strain-dependent occurrence of phosphate solubilization points to a possible functional trait linking vineyard microbiota with raw-material quality and nutrient availability. Importantly, the detection of amino acid decarboxylation in a subset of isolates highlights the need for careful safety assessment when selecting strains for food applications. In this context, isolates lacking detectable decarboxylase activity, such as B21 and B24, may represent candidates for further investigation, although their safety must be confirmed through additional quantitative analyses.

Overall, this work contributes to the understanding of functional diversity and safety-related traits in autochthonous *A. pullulans* populations and identifies candidate strains that may be of interest for future studies. Future research integrating genomics, metabolomics, and validation in real food matrices will be necessary to confirm technological performance, clarify their role in flavor and nutrient modulation, and ensure safe industrial application.

## Figures and Tables

**Table 1:** Enzymatic activities of *Aureobasidium pullulans* isolates, expressed as mean enzymatic index (EI) (±SD) per vineyard or as number of positive isolates/total isolates.

Vineyard	Amylase EI	Cellulase EI	Pectinase EI	Xylanase EI	*β*-Glucosidase (+/−)	Esterase (+/−)	Protease (+/−)	Urease (+/−)
I	1.05 ± 0.07 ^a^	2.55 ± 0.67 ^ab^	3.28 ± 0.43 ^bc^	1.34 ± 0.40 ^a^	7/7 (100%)	7/7 (100%)	7/7 (100%)	4/7 (57%)
P	1.00 ± 0.00 ^a^	3.38 ± 0.99 ^bc^	3.58 ± 0.74 ^bc^	1.80 ± 0.52 ^ab^	2/2 (100%)	2/2 (100%)	2/2 (100%)	0/2 (0%)
DR	1.15 ± 0.16 ^a^	3.92 ± 0.15 ^c^	2.63 ± 0.42 ^a^	3.07 ± 0.20 ^c^	2/2 (100%)	2/2 (100%)	2/2 (100%)	1/2 (50%)
B	1.03 ± 0.05 ^a^	2.71 ± 0.71 ^ab^	3.47 ± 0.41 ^bc^	1.91 ± 0.52 ^ab^	8/9 (89%)	9/9 (100%)	9/9 (100%)	4/9 (44%)
K	1.06 ± 0.05 ^a^	3.29 ± 0.36 ^bc^	3.42 ± 0.51 ^bc^	2.02 ± 0.48 ^ab^	4/4 (100%)	4/4 (100%)	3/4 (75%)	2/4 (50%)
VP	1.04 ± 0.05 ^a^	2.52 ± 0.80 ^ab^	3.78 ± 0.32 ^c^	1.62 ± 0.36 ^ab^	5/7 (71%)	7/7 (100%)	7/7 (100%)	4/7 (57%)
S	1.07 ± 0.08 ^a^	2.93 ± 1.09 ^ab^	3.57 ± 0.86 ^bc^	2.54 ± 1.11 ^bc^	3/10 (30%)	10/10 (100%)	7/10 (70%)	6/10 (60%)
N	1.26 ± 0.27 ^b^	3.01 ± 0.52 ^abc^	3.21 ± 0.43 ^abc^	1.70 ± 0.55 ^ab^	11/12 (92%)	12/12 (100%)	12/12 (100%)	8/12 (67%)
Z	1.07 ± 0.07 ^a^	2.22 ± 0.33 ^a^	3.11 ± 0.16 ^ab^	2.00 ± 0.45 ^ab^	5/5 (100%)	5/5 (100%)	5/5 (100%)	0/5 (0%)
V	1.08 ± 0.05 ^a^	3.43 ± 0.77 ^bc^	3.61 ± 0.41 ^bc^	3.39 ± 1.62 ^c^	8/9 (89%)	9/9 (100%)	9/9 (100%)	3/9 (33%)
O	1.03 ± 0.04 ^a^	3.15 ± 0.62 ^bc^	3.15 ± 0.49 ^abc^	1.73 ± 0.31 ^ab^	3/3 (100%)	3/3 (100%)	3/3 (100%)	1/3 (33%)

Values with different superscript letters within each column are significantly different (*p* < 0.05) according to the Tukey–Kramer’s (HSD) test.

**Table 2:** Screening results of *Aureobasidium pullulans* isolates for phosphate solubilization; biogenic amine production via decarboxylation of the amino acids ornithine, phenylalanine, histidine, and lysine; osmotic and halotolerance; and temperature tolerance at 50 °C (2 and 4 h).

Vineyard	Phosphate Solubilization * (+/−)	Biogenic Amines Production *				Osmotic Tolerance (Glucose) *		Halotolerance **	Temperature Tolerance (50 °C) *	
		Ornithine (+/−)	Phenylalanine (+/−)	Histidine (+/−)	Lysine (+/−)	30% (+/−)	50% (+/−)	NaCl (%)	2 h (+/−)	4 h (+/−)
I	1/7 (14%)	7/7 (100%)	7/7 (100%)	7/7 (100%)	0/7 (0%)	7/7 (100%)	7/7 (100%)	14.29 ± 0.76 ^d^	0/7 (0%)	0/7 (0%)
P	0/2 (0%)	0/2 (0%)	1/2 (50%)	2/2 (100%)	0/2 (0%)	2/2 (100%)	2/2 (100%)	14.00 ± 0.00 ^d^	0/2 (0%)	0/2 (0%)
DR	0/2 (0%)	1/2 (50%)	2/2 (100%)	2/2 (100%)	0/2 (0%)	2/2 (100%)	2/2 (100%)	13.00 ± 1.41 ^c,d^	0/2 (0%)	0/2 (0%)
B	2/9 (22%)	7/9 (78%)	6/9 (67%)	6/9 (67%)	0/9 (0%)	9/9 (100%)	9/9 (100%)	14.00 ± 0.00 ^d^	2/9 (22%)	0/9 (0%)
K	2/4 (50%)	2/4 (50%)	2/4 (50%)	4/4 (100%)	0/4 (0%)	4/4 (100%)	4/4 (100%)	13.00 ± 2.00 ^c,d^	0/4 (0%)	0/4 (0%)
VP	0/7 (0%)	6/7 (86%)	6/7 (86%)	3/7 (43%)	0/7 (0%)	7/7 (100%)	7/7 (100%)	13.14 ± 1.57 ^c,d^	0/7 (0%)	0/7 (0%)
S	4/10 (40%)	5/10 (50%)	7/10 (70%)	8/10 (80%)	0/10 (0%)	10/10 (100%)	8/10 (80%)	10.20 ± 2.39 ^a,b^	1/10 (10%)	0/10 (0%)
N	1/12 (8%)	10/12 (83%)	10/12 (83%)	10/12 (83%)	0/12 (0%)	12/12 (100%)	10/12 (83%)	11.83 ± 0.58 ^b,c^	5/10 (50%)	0/10 (0%)
Z	3/5 (60%)	3/5 (60%)	5/5 (100%)	2/5 (40%)	0/5 (0%)	5/5 (100%)	5/5 (100%)	10.00 ± 0.00 ^a^	1/5 (20%)	0/5 (0%)
V	8/9 (89%)	8/9 (89%)	6/9 (67%)	9/9 (100%)	0/9 (0%)	9/9 (100%)	8/9 (89%)	10.00 ± 0.00 ^a^	0/9 (0%)	0/9 (0%)
O	1/3 (33%)	2/3 (67%)	3/3 (100%)	1/3 (33%)	0/3 (0%)	3/3 (100%)	3/3 (100%)	10.89 ± 2.03 ^a,b^	1/3 (33%)	0/3 (0%)

* Data are expressed as the number of positive isolates relative to the total isolates examined, followed by the corresponding percentage. ** Values are expressed as the mean ± standard deviation. Different superscript letters within each column are significantly different (*p* < 0.05) according to the Tukey–Kramer’s (HSD) test.

## Data Availability

The original contributions presented in this study are included in this article/Supplementary Materials. Further inquiries can be directed to the corresponding author.

## Supplementary Materials

The following supporting information can be downloaded at: https://www.mdpi.com/article/10.3390/foods15091573/s1, Supplementary Table S1: Enzymatic activities of the *Aureobasidium pullulans* isolates.; Supplementary Table S2: Screening of *Aureobasidium pullulans* isolates for phosphate solubilization; biogenic amine production via decarboxylation of the amino acids ornithine, phenylalanine, histidine, and lysine; and tolerance to challenging environmental conditions, including salt and osmotic stress, elevated temperature, and varying pH.

## Abbreviations

The following abbreviations are used in this manuscript:

ND	Northern Dalmatia
DH	Dalmatian Hinterland
CSD	Central and Southern Dalmatia
BLAST	Basic Local Alignment Search Tool
YPD	Yeast extract–Peptone–Dextrose
WL	Wallerstein Laboratory differential agar
MEA	Malt Extract Agar
CMC	CarboxyMethyl Cellulose
PDA	Potato Dextrose Agar
NA	Nutrient Agar
EI	Enzymatic Index
ANOVA	One-way analysis of variance
BA	Biogenic Amine
