# Supplementary material for: Food Biotechnology Potential of Grape-Derived Aureobasidium pullulans: Characterization and Screening for Enzyme Production Capacity
Source: Foods. 2026 May 3;15(9):1573. doi: 10.3390/foods15091573 (PMC13164108; doi:10.3390/foods15091573)
Supplement: Supplementary file 1 [file foods-15-01573-s001.zip › Supplementary Table S1.pdf]

Supplementary Table S1. Enzymatic activities of the *Aureobasidium pullulans* isolates.

Vineyard	Isolate	Amylase EI	Cellulase EI	Pectinase EI	Xylanase EI	*β*-glucosidase (+/-)	Esterase (+/-)	Protease (+/-)	Urease (+/-)
I	I-4	1.00±0.00	2.50±0.26	2.92±0.08	1.00±0.00	+	+	+	+
	I-7	1.00±0.00	1.82±0.03	2.82±0.10	1.08±0.04	+	+	+	+
	I-17	1.03±0.01	2.50±0.10	3.00±0.13	1.29±0.07	+	+	+	-
	I-18	1.18±0.02	2.38±0.07	3.75±0.05	1.42±0.03	+	+	+	+
	I-19	1.00±0.00	2.11±0.10	3.20±0.09	1.32±0.08	+	+	+	-
	I-20	1.14±0.01	4.00±0.20	4.00±0.05	2.22±0.05	+	+	+	-
	I-26	1.00±0.00	2.57±0.08	3.30±0.10	1.06±0.02	+	+	+	+
P	P-9	1.00±0.00	4.25±0.03	4.25±0.05	2.27±0.01	+	+	+	-
	P-10	1.00±0.00	2.50±0.36	2.91±0.16	1.33±0.02	+	+	+	-
DR	DR-5	1.29±0.02	4.00±0.06	2.27±0.08	3.25±0.03	+	+	+	-
	DR-9	1.00±0.00	3.80±0.10	3.00±0.17	2.89±0.07	+	+	+	+
B	B-12	1.00±0.00	2.11±0.04	3.22±0.14	2.50±0.08	-	+	+	-
	B-15	1.10±0.00	3.33±0.05	3.18±0.06	2.31±0.04	+	+	+	-
	B-16	1.00±0.00	2.71±0.08	3.04±0.14	1.23±0.04	+	+	+	-
	B-17	1.00±0.00	2.38±0.09	3.44±0.14	1.36±0.01	+	+	+	-
	B-18	1.00±0.00	2.22±0.11	3.56±0.13	2.50±0.13	+	+	+	-
	B-21	1.12±0.03	4.00±0.10	3.76±0.07	1.67±0.02	+	+	+	+
	B-24	1.00±0.00	1.70±0.02	4.26±0.06	1.79±0.04	+	+	+	+
	B-25	1.00±0.00	3.40±0.10	3.00±0.17	2.50±0.05	+	+	+	+
	B-29	1.08±0.01	2.50±0.05	3.76±0.07	1.38±0.01	+	+	+	+
K	K-3	1.03±0.02	3.40±0.05	3.67±0.06	1.82±0.03	+	+	-	+
	K-12	1.04±0.02	3.00±0.02	2.59±0.11	1.43±0.01	+	+	+	+
	K-28	1.14±0.02	3.80±0.10	3.78±0.03	2.17±0.06	+	+	+	-
	K-32	1.04±0.04	2.97±0.06	3.65±0.16	2.67±0.05	+	+	+	-
VP	VP-4	1.09±0.02	2.11±0.04	3.60±0.02	1.36±0.04	+	+	+	+
	VP-5	1.00±0.00	2.40±0.20	3.37±0.15	1.47±0.07	+	+	+	-
	VP-6	1.00±0.00	2.11±0.06	3.78±0.10	2.00±0.02	-	+	+	+
	VP-7	1.00±0.00	1.67±0.10	4.00±0.09	1.00±0.00	+	+	+	+
	VP-9	1.03±0.01	4.00±0.10	3.58±0.03	1.80±0.13	+	+	+	-
	VP-24	1.00±0.00	2.00±0.13	3.78±0.03	1.69±0.15	+	+	+	-
	VP-27	1.13±0.05	3.33±0.16	4.38±0.14	2.00±0.11	-	+	+	+
S	S-2	1.18±0.02	4.20±0.10	5.38±0.12	3.76±0.12	+	+	+	+
	S-3	1.15±0.02	5.33±0.09	3.71±0.10	2.38±0.01	-	+	+	+
	S-4	1.00±0.00	2.00±0.05	2.71±0.04	4.80±0.05	-	+	+	+
	S-5	1.00±0.00	2.22±0.13	4.29±0.24	1.54±0.03	+	+	+	-
	S-6	1.20±0.02	2.00±0.05	4.33±0.09	2.94±0.01	-	+	-	+
	S-7	1.00±0.00	2.57±0.11	3.60±0.07	1.11±0.02	+	+	-	-
	S-13	1.00±0.00	2.67±0.02	3.00±0.15	2.67±0.04	-	+	+	-
	S-15	1.00±0.00	2.13±0.12	2.90±0.05	1.38±0.02	-	+	+	+
	S-27	1.10±0.05	3.80±0.05	3.22±0.05	3.00±0.20	-	+	+	-
	S-30	1.08±0.00	2.38±0.06	2.52±0.08	1.82±0.02	-	+	-	+
N	N-1	2.00±0.15	3.40±0.05	2.50±0.03	1.78±0.06	+	+	+	-
	N-3	1.06±0.03	2.33±0.16	2.89±0.05	1.33±0.03	+	+	+	+
	N-4	1.03±0.03	2.50±0.05	3.71±0.19	1.20±0.05	+	+	+	-
	N-12	1.40±0.10	3.17±0.08	3.63±0.06	1.54±0.03	+	+	+	-
	N-13	1.09±0.04	2.92±0.07	3.20±0.08	1.67±0.08	+	+	+	+
	N-15	1.09±0.06	3.20±0.05	3.16±0.09	1.41±0.05	-	+	+	+
	N-16	1.32±0.08	2.50±0.10	2.78±0.07	2.50±0.06	+	+	+	+
	N-17	1.46±0.08	3.60±0.09	3.30±0.19	3.13±0.02	+	+	+	-
	N-18	1.08±0.05	3.60±0.10	3.33±0.04	1.23±0.03	+	+	+	+
	N-21	1.22±0.01	3.80±0.00	4.00±0.13	1.50±0.03	+	+	+	+
	N-23	1.17±0.06	2.33±0.14	2.73±0.04	1.50±0.02	+	+	+	+
	N-26	1.14±0.01	2.80±0.20	3.33±0.02	1.85±0.09	+	+	+	+
Z	Z-2	1.06±0.02	1.89±0.20	2.90±0.05	2.27±0.03	+	+	+	-
	Z-5	1.12±0.04	2.33±0.06	3.33±0.09	1.67±0.09	+	+	+	-
	Z-9	1.17±0.04	2.33±0.17	3.10±0.09	1.79±0.09	+	+	+	-
	Z-13	1.00±0.00	2.67±0.14	3.05±0.02	2.71±0.03	+	+	+	-
	Z-20	1.00±0.00	1.89±0.04	3.16±0.08	1.55±0.03	+	+	+	-

Vineyard	Isolate	Amylase EI	Cellulase EI	Pectinase EI	Xylanase EI	*β*-glucosidase (+/-)	Esterase (+/-)	Protease (+/-)	Urease (+/-)
V	V-1	1.10±0.0.1	3.00±0.09	3.88±0.01	1.35±0.10	+	+	+	+
	V-2	1.14±0.04	3.50±0.17	4.29±0.14	3.50±0.03	+	+	+	-
	V-5	1.03±0.01	4.00±0.24	3.00±0.02	1.35±0.04	+	+	+	-
	V-6	1.12±0.04	4.20±0.20	3.30±0.06	1.47±0.05	-	+	+	-
	V-7	1.11±0.05	4.20±0.17	4.00±0.13	3.86±0.07	+	+	+	-
	V-8	1.02±0.01	2.00±0.10	3.76±0.02	4.40±0.02	+	+	+	+
	V-11	1.04±0.01	2.50±0.26	3.75±0.07	3.57±0.05	+	+	+	-
	V-12	1.11±0.04	4.00±0.17	3.20±0.01	6.00±0.07	+	+	+	-
	V-30	1.13±0.03	3.50±0.00	3.37±0.02	5.00±0.02	+	+	+	+
O	O-14	1.00±0.00	2.37±0.03	3.41±0.07	1.41±0.22	+	+	+	-
	O-15	1.00±0.00	3.40±0.36	2.52±0.14	1.71±0.02	+	+	+	+
	O-22	1.07±0.01	3.67±0.12	3.53±0.02	2.07±0.04	+	+	+	-

EI, enzymatic index; +, -, presence or absence of activity.
