# Supplementary material for: Food Biotechnology Potential of Grape-Derived Aureobasidium pullulans: Characterization and Screening for Enzyme Production Capacity
Source: Foods. 2026 May 3;15(9):1573. doi: 10.3390/foods15091573 (PMC13164108; doi:10.3390/foods15091573)
Supplement: Supplementary file 1 [file foods-15-01573-s001.zip › Supplementary Table S2.pdf]

Supplementary Table S2. Screening of *Aureobasidium pullulans* isolates for phosphate solubilization; biogenic amine production via decarboxylation of the amino acids ornithine, phenylalanine, histidine, and lysine; and tolerance to challenging environmental conditions, including salt and osmotic stress, elevated temperature, and varying pH.

Vineyard	Isolate	Phosphate solubilization (+/-)	Biogenic amines production				Osmotic tolerance (glucose)		Halotolerance	Temperature tolerance (50°C)		pH tolerance	
			Ornithine (+/-)	Phenylalanine (+/-)	Histidine (+/-)	Lysine (+/-)	30% (+/-)	50% (+/-)	NaCl (%)	2h (+/-)	4h (+/-)	Min	Max
I	I-4	-	+	+	+	-	+	+	14	-	-	4	11
	I-7	-	+	+	+	-	+	+	14	-	-	4	11
	I-17	-	+	+	+	-	+	+	14	-	-	4	11
	I-18	-	+	+	+	-	+	+	14	-	-	4	11
	I-19	-	+	+	+	-	+	+	16	-	-	4	11
	I-20	+	+	+	+	-	+	+	14	-	-	4	11
	I-26	-	+	+	+	-	+	+	14	-	-	4	11
P	P-9	-	-	-	+	-	+	+	14	-	-	4	11
	P-10	-	-	+	+	-	+	+	14	-	-	4	11
DR	DR-5	-	-	+	+	-	+	+	12	-	-	4	11
	DR-9	-	+	+	+	-	+	+	14	-	-	4	11
B	B-12	-	+	+	+	-	+	+	14	-	-	4	11
	B-15	-	+	+	+	-	+	+	14	-	-	4	11
	B-16	-	+	+	+	-	+	+	14	-	-	4	11
	B-17	-	+	+	+	-	+	+	14	-	-	4	11
	B-18	-	+	+	+	-	+	+	14	+	-	4	11
	B-21	+	-	-	-	-	+	+	14	-	-	4	11
	B-24	-	-	-	-	-	+	+	14	+	-	4	11
	B-25	-	+	-	+	-	+	+	14	-	-	4	11
	B-29	+	+	+	-	-	+	+	14	-	-	4	11
K	K-3	-	-	+	+	-	+	+	14	-	-	4	11
	K-12	+	+	-	+	-	+	+	10	-	-	4	7
	K-28	+	+	-	+	-	+	+	14	-	-	4	11
	K-32	-	-	+	+	-	+	+	14	-	-	4	11
VP	VP-4	-	+	+	+	-	+	+	14	-	-	4	11
	VP-5	-	-	+	+	-	+	+	14	-	-	4	11
	VP-6	-	+	+	-	-	+	+	14	-	-	4	11
	VP-7	-	+	+	-	-	+	+	10	-	-	4	11
	VP-9	-	+	+	-	-	+	+	12	-	-	4	11
	VP-24	-	+	-	-	-	+	+	14	-	-	4	11
	VP-27	-	+	+	+	-	+	+	14	-	-	4	11
S	S-2	-	+	+	+	-	+	+	12	-	-	4	11
	S-3	-	-	+	+	-	+	+	10	-	-	4	11
	S-4	-	-	+	+	-	+	-	6	-	-	4	11
	S-5	-	+	+	+	-	+	+	12	-	-	4	11
	S-6	-	+	+	+	-	+	+	12	-	-	4	11
	S-7	+	-	-	+	-	+	+	12	+	-	4	11
	S-13	+	-	-	-	-	+	+	10	-	-	4	11
	S-15	-	+	+	-	-	+	+	10	-	-	7	9
	S-27	+	+	-	+	-	+	+	12	-	-	4	11
	S-30	+	-	+	+	-	+	-	6	-	-	4	11
N	N-1	-	+	+	+	-	+	-	10	-	-	4	11
	N-3	-	+	+	+	-	+	+	12	-	-	4	11

Vineyard	Isolate	Phosphate solubilization (+/-)	Biogenic amines production				Osmotic tolerance (glucose)		Halotolerance	Temperature tolerance (50°C)		pH tolerance	
			Ornithine (+/-)	Phenylalanine (+/-)	Histidine (+/-)	Lysine (+/-)	30% (+/-)	50% (+/-)	NaCl (%)	2h (+/-)	4h (+/-)	Min	Max
	N-4	-	-	+	+	-	+	-	12	-	-	4	11
	N-12	+	-	+	+	-	+	+	12	-	-	4	11
	N-13	-	+	+	+	-	+	+	12	-	-	4	11
	N-15	-	+	+	-	-	+	+	12	+	-	4	11
N	N-16	-	+	-	+	-	+	+	12	+	-	4	11
	N-17	-	+	-	-	-	+	+	12	+	-	4	11
	N-18	-	+	+	+	-	+	+	12	+	-	4	11
	N-21	-	+	+	+	-	+	+	12	+	-	4	11
	N-23	-	+	+	+	-	+	+	12	-	-	4	11
	N-26	-	+	+	+	-	+	+	12	-	-	4	11
	Z-2	-	-	+	+	-	+	+	10	-	-	4	11
	Z-5	+	-	+	+	-	+	+	10	-	-	4	11
Z	Z-9	-	+	+	-	-	+	+	10	+	-	4	11
	Z-13	+	+	+	-	-	+	+	10	-	-	4	11
	Z-20	+	+	+	-	-	+	+	10	-	-	4	11
	V-1	+	+	+	+	-	+	-	6	-	-	4	11
	V-2	+	+	+	+	-	+	+	10	-	-	4	11
	V-5	-	+	+	+	-	+	+	12	-	-	4	11
	V-6	+	+	+	+	-	+	+	12	-	-	4	11
V	V-7	+	+	-	+	-	+	+	12	-	-	4	11
	V-8	+	+	-	+	-	+	+	12	-	-	4	11
	V-11	+	+	-	+	-	+	+	10	-	-	4	11
	V-12	+	-	+	+	-	+	+	12	-	-	4	11
	V-30	+	+	+	+	-	+	+	12	-	-	4	11
	O-14	-	+	+	-	-	+	+	10	-	-	4	11
O	O-15	-	+	+	-	-	+	+	10	+	-	4	11
	O-22	+	-	+	+	-	+	+	10	-	-	4	11

+, -, presence or absence of activity.
